# Effect of Isolated Hip Musculature Strengthening Program in Avascular Necrosis: A Case Report

**DOI:** 10.7759/cureus.30360

**Published:** 2022-10-16

**Authors:** Shrutika M Mungal, Pooja Dhage, Nikita S Deshmukh

**Affiliations:** 1 Physiotherapy, Ravi Nair Physiotherapy College, Datta Meghe Institute of Medical Sciences, Wardha, IND; 2 Musculoskeletal Physiotherapy, Ravi Nair Physiotherapy College, Datta Meghe Institute of Medical Sciences, Wardha, IND; 3 Musculoskeletal physiotherapy, Ravi Nair Physiotherapy College, Datta Meghe Institute of Medical Sciences, Wardha, IND

**Keywords:** muscle energy technique (met), group muscle strengthening, pain, physiotherapy, avascular necrosis of the femoral head

## Abstract

Avascular necrosis is the destruction of bone tissue as a result of a lack of blood flow. Alternative synonyms for this condition include osteonecrosis, aseptic necrosis, and ischemic bone necrosis. In dire cases, bone collapse may occur. It tends to affect the hip, shoulder, knee, hand, and foot. Other typical locations are the shoulder, knees, and ankles. Avascular necrosis is a clinical condition characterized by complaints such as localized discomfort and limited range of motion. Early conservative treatment helps to decrease the progression of the symptoms. In the initial stages of avascular necrosis, physiotherapy plays a crucial role to reduce pain and improve the range of motion. This case study aims to inform readers about the conservative treatment protocol that can manage avascular necrosis in the initial condition.

## Introduction

Avascular necrosis of the femoral head is a rare disease that is marked by apoptosis of osseous cells - bone marrow, bone-forming, and bone-destroying cells - that results in bone implosion with eventual involvement of the overlying cartilage exacerbating thinning of the surface of the head and ultimately resulting in secondary osteoarthritis [1]. The bone structures collapse, resulting in pain, joint dysfunction, and long-term joint damage. The epiphysis (the terminal part of a long bone), such as the femoral and humeral heads and femoral condyles, is most typically affected by avascular necrosis, although smaller bones can also be damaged. Avascular necrosis is commonly encountered in the femoral head in clinical practice [2]. Non-traumatic avascular necrosis of the femoral head (NONFH) is a degenerative disease that develops at the femoral head due to a blood supply impairment. This persistent, complex, and disabling illness can cause severe morbidity in people of any age group. Despite well-defined clinical symptoms, the origin and epidemiology of NONFH remain unknown [3]. Steroids have been proven to enhance the likelihood of non-site-specific osteonecrosis. NONFH is essentially bone cell death due to decreased microvascular circulation, which is hypothesized to be induced by mechanical vascular disruption, intravascular occlusion, and extravascular compression. These variables have been linked to an increased risk of avascular necrosis: high blood triglyceride levels, low total cholesterol non-high-density lipoprotein cholesterol and high-density lipoprotein cholesterol, male gender, urban habitation, family history of avascular necrosis, heavy smokers, abuse of alcohol, overweight, coagulopathies, vasculopathy, human immune deficiency virus, extensive use of steroids, chemotherapy, and immunosuppressive drugs [4]. Ficat and Arlet's grading system for avascular necrosis is based on clinical complaints, radiographic abnormalities, and bone scan uptake. Stage 0 is a preclinical stage with no abnormalities in radiographs other than reduced activation on a bone scan. Stage I is differentiated by clinical symptoms and higher uptake on a bone scan. In stage II, sclerotic or cystic abnormalities on radiographs are seen. In stage III the radiographic crescent sign with joint space is preserved, whereas femoral head flattening and hip joint arthritic anomalies are present in stage IV [5].

The gluteus medius is the primary hip abductor. This muscle's concentric activity governs the adduction action of the hip with body weight-bearing activities. As a result of gluteus medius insufficiency, excessive hip adduction may occur during one limb body weight-bearing activities [6]. The muscle energy technique (MET) is a manual technique in which the physical therapist does not control the amount of force; the patient’s voluntary contractions would lead to an increase in variable intensity. MET, along with post-isometrics such as proprioceptive neuromuscular facilitation (PNF), is more effective than static stretching at improving the flexibility of tightened muscles [7]. As a result, it is a manual treatment to restore any limited range of motion. This technique can improve muscle contracture or weakening, which reduces localized edema by triggering rhythmic movement. After this, the patient can perform an isometric contraction and a post-isometric relaxation of the contracted muscle. This behavior is triggered by the Golgi tendon organ [7]. When using MET to lengthen shortened muscles, the following phases are followed: stretch the hamstrings to a palpable “barrier,” voluntary isometric hamstring muscle contractions during stretch against the proportionate counterforce, the muscle is let to relax whereas the therapist maintains a stretch for a specified time period, and the therapist picks up the first barrier following relaxation so that the muscle has been extended to a new barrier [8].

In extreme situations, pain becomes chronic and spreads to nearby tissues such as the thigh and buttocks. The cause may be traumatic or non-traumatic. If not addressed earlier, it might lead to major complications such as atrial insufficiency, deep vein thrombosis, and pulmonary embolism leading to surgery. X-ray investigation aid in the identification of the damaged portion and MRI findings for the conforming stage of the condition. Physical therapy helps in the treatment by producing symptomatic relief and improving functional independence. This case study is about a male who complained about pain in his right hip joint and difficulty walking due to the condition and was diagnosed with stage III avascular necrosis of the right femoral head on observation of gait, hip deformity, groin tenderness, restricted range of motion of the hip joint, and limb length. Special tests such as the flexion abduction external rotation test (FABER) and the Trendelenburg test were positive.

## Case presentation

Patient information

**Figure 1:**
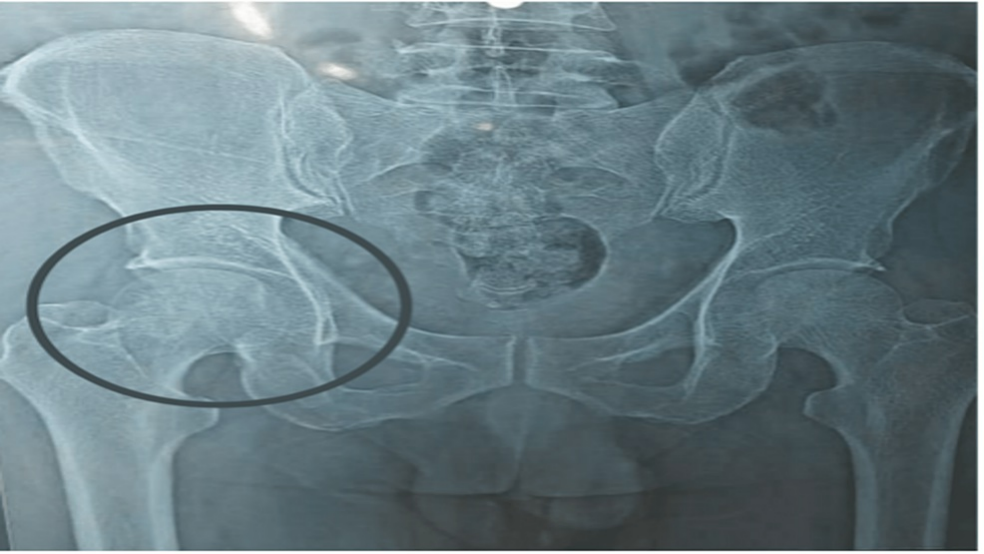
Anteroposterior pelvic radiograph showing sclerosis or cystic abnormalities in the femoral head. The joint space was protected.

A 42-year male patient, a coal miner by occupation with right-hand dominance, presented to the hospital with a chief complaint of pain and decreased range of motion in the right lower limb for two months. The patient was apparently all right for a few months when he started to experience discomfort in his right hip. The patient had a history of using oral COVID steroids for 25 days one year back for the management of COVID symptoms and had a history of the COVID vaccination three months prior to presentation. The pain was gradual in onset and progressive in nature, with a numerical pain rating scale intensity of 7/10 while movement and 5/10 during rest (visual analog scale). It was a dull aching that worsened with movement and activity and was eased by rest and medicine. The pain worsened with time, and the patient found it difficult to sit cross-legged or squat. The patient went to the general practitioner and was prescribed sodium diclofenac for pain treatment; however, no alleviation was found, so he chose to visit the orthopedics outpatient department with complaints of pain in his right hip and difficulty walking. The orthopedic surgeon recommended an X-ray, which revealed avascular necrosis in the right femoral head grade II according to Ficat and Arlet classification (Figure 1). Physiotherapy was referred for the right hip due to restricted or limited range of motion.

Clinical findings

Written consent was taken from the patient. The vitals were normal on general examination. He was afebrile, with a pulse rate of 84 beats/minute, respiratory rate of 20 breaths/minute, blood pressure of 126/80 mmHg, and body mass index of 28.04 kg/m2. The underlying skin appeared to be normal on examination. The patient showed hip hiking and decreased adduction of the right hip. The patient had a history of COVID steroids a year ago. The local temperature was not increased on inspection, and grade 2 pain was noted above the right anterior joint line. The range of motion of the right (affected side) hip was considerably restricted and painful in all ranges pre-treatment (Table 1). Muscle weakening was seen in the right lower leg (pre-treatment) (Table 2).

**Table 1:** ROM pre-treatment assessment in degree (right hip) ROM, range of motion

Motion	Pre-assessment active ROM	Pre-assessment passive ROM
Hip flexion	0-30	0-35
Hip extension	17-0	23-0
Hip abduction	0-25	0-28
Hip adduction	0-20	0-25
Hip lateral rotation	0-25	0-25
Hip medial rotation	0-20	0-20

**Table 2:** Muscle manual testing pre-assessment

Motions	Right leg pre-assessment
Hip flexion	2/5
Hip extension	3/5
Hip abduction	2/5
Hip adduction	3/5
Hip internal rotation	3/5
Hip external rotation	3/5
Knee flexion	3/5
Knee extensors	3/5

**Figure 2:**
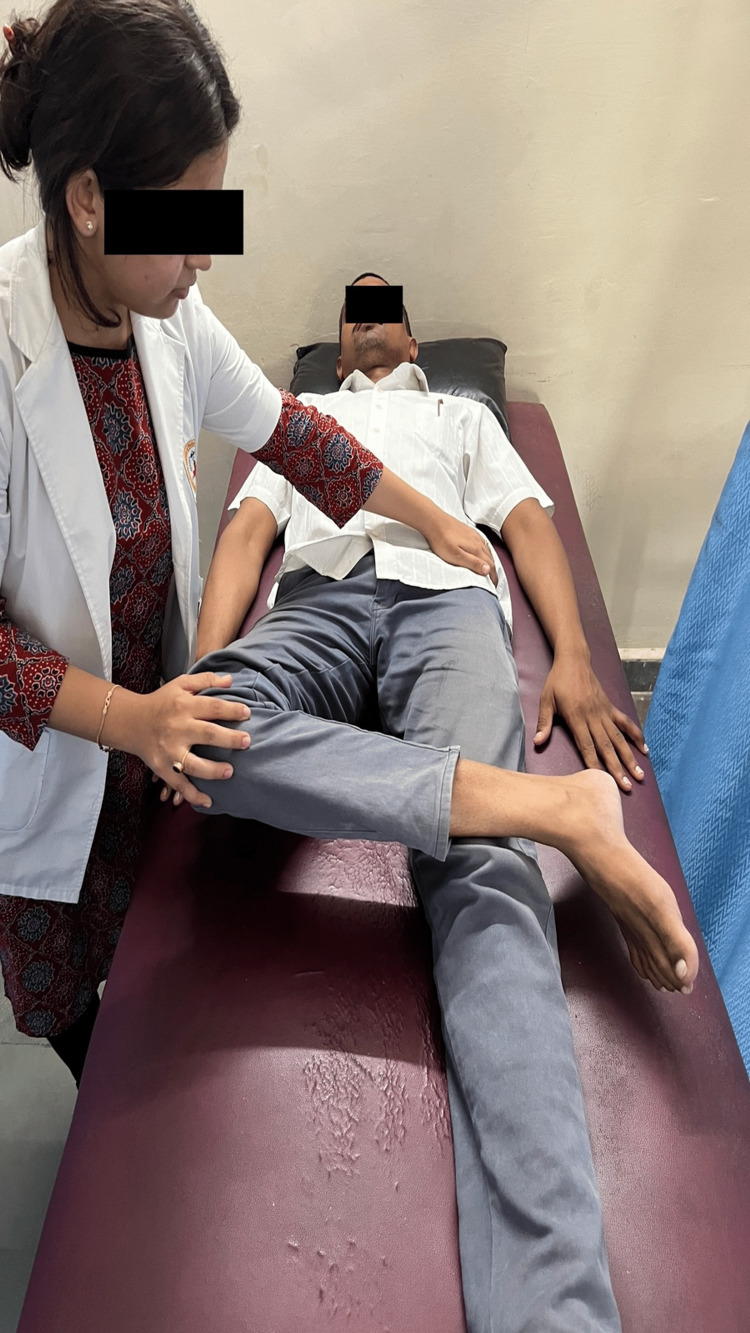
Flexion abduction external rotation test showing abductor muscle tightness

The left thigh muscle also exhibited symptoms of atrophy. The afflicted side's abductor (gluteus medius, gluteus minimus, tensor fascia late) and hamstring muscles were tight, and special tests such as 90-90 hamstrings and FABER were positive (Figure 2). The circulation was intact to the distal extremity. The affected side's abductor and hamstring muscles were tight. During the neurological assessment, both sides showed normal reflexes and sensory evaluation. Trendelenburg's sign was positive. On gait examination, an altered gait pattern was seen, in which the stance phase and foot flat was reduced.

Physiotherapy intervention

The protocol given in Table 3 was followed for two weeks, which helped the patient post-treatment improve in the range of motion of the hip joint in two weeks (Table 4) and muscle strength (Table 5).

**Table 3:** Physiotherapy rehabilitation

Days	Treatment	Rationale
0-2 days	Hip flexors and abductors of the right hip of the group muscle (10 repetitions and 3 sets) [9]. Heel slides (10 repetitions and 3 sets). Ultrasound: mode-continuous, frequency of 1 MHz, transducer head of 0.8 mm, duration of 6 min [10].	To reduce muscular atrophy while maintaining muscle contractility. To improve circulation, mobility, and flexibility. Decreases muscular spasms, pain, and chronic inflammation by increasing blood flow.
2-5 days	Hip flexors and abductors of the right hip of the group muscle with resistance sandbag (1 kg) (10 repetitions with 5-sec hold and 3 sets). Hamstrings stretch (muscle energy technique)	To improve muscle strength and endurance. It helps in recovering mobility, reducing tissue edema, reducing muscular spasms, stretching fibrous tissue, and retraining the inter-segmentally related muscles stabilizing function [11].
5-7 days	Hip flexors and abductors of the right hip of the group muscle with resistance sandbag (5 kg) (15 repetitions with 10-sec hold and 5 sets).	To regain and maintain the range of joints.
1-2 weeks	Hamstring stretch (muscle energy technique) measuring with the goniometer. While standing: hip flexors and abductors with thera-bands (15 repetitions with 15-sec hold and 5 sets)	To improve the strength of the hamstrings, to improve the strength of hip flexors and abductors, and to correct hip hiking.

**Table 4:** Post-treatment assessment in degree (right hip) ROM, range of motion

Motion	Post-assessment active ROM	Post-assessment passive ROM
Hip flexion	0-100	0-117
Hip extension	28-0	30-0
Hip abduction	0-35	0-35
Hip adduction	0-30	0-30
Hip lateral rotation	0-35	0-35
Hip medial rotation	0-38	0-38

**Table 5:** Muscle manual testing post-assessment

Motions	Right leg post-assessment
Hip flexion	4/5
Hip extension	4/5
Hip abduction	4/5
Hip adduction	4/5
Hip internal rotation	3/5
Hip external rotation	3/5
Knee flexion	4/5
Knee extensors	4/5

## Discussion

Studies on static, dynamic, and PNF stretching techniques on extensibility state that active stretching, such as ballistic and PNF, requires people to contract their muscles voluntarily. PNF stretches involve antagonist contraction followed by relaxation (CR). They can also use contraction of the agonist of the stretched muscle followed by relaxation (contract-relax, antagonist-contract [CRAC]) [12]. A review conducted on METs in the rehabilitation therapy of acute and chronic non-specific neck pain found that MET is beneficial in the treatment of both acute and chronic neck pain. Good results were seen by combining the MET with conventional therapy, which included the use of modalities such as transcutaneous electrical muscle stimulator, ultrasounds, or thermotherapy to relieve symptoms and advise the patient on cervical muscle stretching, strengthening exercises of the same muscle group, and postural correction during the day-to-day activities to prevent the pain in the condition [13]. The muscles usually have a resting tone that increases musculoskeletal discomfort due to muscle spasms. More sarcomeres form cross bridges and are used in maintaining the increase in active tone as the resting muscle tone increases, while few sarcomeres are available to trigger voluntary contraction. MET leads to autogenic and reciprocal inhibition of the neck muscles, in which one group of muscles get contracted and other gets lengthened. As a result, METs provide an increase in isometric muscle strength [14]. Due to their eccentric function, high tensile forces are exerted on the hamstrings. During the first swing, eccentric and concentric hamstring movements are required for knee and hip flexion. Throughout the phase of the swing, the hamstrings continue to inhibit knee extension with the hip extension. The hamstrings and gluteal together stabilize, decelerate, and accelerate the hip. During the propulsion phase, the medial hamstrings help to decelerate the hip's external rotation [15].

## Conclusions

In terms of pain relief and improvement in range of motion, the use of MET and strengthening program has been recommended. In this case study, we found that an isolated hip muscle strengthening program was effective. The patient’s previous report stated that he had a history of COVID steroids for a period of 25 days and COVID vaccine three months prior to presentation. The quick start of avascular necroses followed by recovery from COVID-19 infection cannot be entirely attributed to steroid therapy and that another positive COVID-19 infection induced factor, it is clear that high dose steroid therapy results in avascular necrosis. Therefore, in the study, we found that the physiotherapy program was effective.
